# Effective cervical cancer prevention in sub-Saharan Africa needs the inclusion of men as key stakeholders

**DOI:** 10.3389/fonc.2025.1509685

**Published:** 2025-01-21

**Authors:** Mathias Dzobo, Tafadzwa Dzinamarira

**Affiliations:** School of Health Systems and Public Health, Faculty of Health Sciences, University of Pretoria, Pretoria, South Africa

**Keywords:** cervical cancer, prevention, men, inclusion, sub-Saharan Africa

## Summary

Cervical cancer is a major public health problem in low-middle-income countries, especially in sub-Saharan Africa. Strategies for cervical cancer prevention are multi-sectorial, often involving many stakeholders. However, male engagement is still not fully embraced. Involving men in cervical cancer prevention can potentially increase access to prevention services and promote health equity among women. Men’s involvement can overcome barriers to women’s access to preventive services and increase community awareness of cervical cancer prevention.

## Opinion

Cervical cancer presents a significant public health challenge globally, particularly in low-middle-income countries where the burden of the disease is highest. According to the World Health Organisation, 19 out of the 20 countries with the highest cervical cancer burden are in sub-Saharan Africa (SSA) (1). Although cervical cancer affects women, involving men in prevention efforts can unlock avenues to increase access to prevention services and promote health equity amongst women in SSA. The sociocultural, economic and political structures of most SSA communities are predominantly male-dominated; therefore, involving men as critical stakeholders is essential to promoting healthcare access and achieving health equity for women (2).

The involvement of men in cervical cancer prevention is a departure from the gendered approaches of cervical cancer prevention that have directed behavioural change, education and awareness activities primarily on women. Men can feel empowered by recognising them as critical key stakeholders, advocates, allies, and partners. Studies in Africa have reported that where men were involved, the uptake of cervical cancer screening services and linkage to care was high, with a corresponding reduction in loss to follow-up on women eligible for treatment (3, 4). Men’s involvement can potentially overcome some of the barriers that have been cited as hindering women’s access to and utilisation of preventive services, such as men’s unwillingness to have their spouses examined by a male health worker and the lack of emotional and financial support for the use of preventive services such as screening (5). The lack of education and awareness by men has been identified in the literature as the most crucial reason for their failure to recognise the importance of cervical cancer prevention efforts as well as the need to offer resources and support towards prevention efforts such as vaccination, early screening and treatment (6).

Central to engaging men as key stakeholders is education to empower them and dispel myths and misconceptions regarding cervical cancer prevention. For instance, education on cervical cancer causes, screening and treatment are essential to raise awareness among men to ensure their support for HPV vaccination, screening of their spouses and treatment and further management (7). Myths regarding the link between cervical cancer and promiscuity must be dispelled to ensure that women do not fear reporting screening results to their spouses. Additionally, men need to be educated on the need to offer support during women’s treatment, which requires a period of sexual abstinence; in one study, men were not willing to abstain during women’s treatment and would either forcefully have sex or seek sex elsewhere, prompting their women to default on treatment. It is paramount to design strategies for disseminating information to men. A study conducted in Uganda recommended using radio communication to reach men and including men in edutainment initiatives to reach other men with cervical cancer prevention messages and information (4). By educating and leveraging men’s societal influence and power, men can disseminate information about cervical cancer prevention, debunk myths, and emphasise the importance of vaccination, early screening and treatment. Their involvement significantly enhances community awareness.

Although strides have been made in promoting gender equality, it is evident that men still occupy the critical social, economic, and political structures of many African societies, consequently relegating sexual health decision-making to men. An all-inclusive approach is critical as it can ride on men’s existing power in society to push for more equitable resource distribution and increase awareness and advocacy for cervical cancer prevention. This is particularly important in rural and remote areas where communal leaders and men largely control the means of production, and women are primarily unemployed and rely exclusively on men for financial support for the welfare of the family and for seeking health services. Existing cultural beliefs and gender roles in rural societies hinder women’s access to services such as cervical cancer screening and treatment (8).

Men’s involvement in cervical cancer prevention extends beyond awareness and advocacy. Any effective public health prevention programme requires financial support for sustainability. From the grassroots to the communal level to the highest decision-making bodies in non-governmental and government organisations, men constitute the majority of the decision-making structures, directly impacting resource allocation and distribution. Therefore, reaching men is critical to ensure that there are adequate resources for implementing cervical cancer prevention programmes and ensuring their sustainability.

Cervical cancer prevention efforts in low-resource settings such as the SSA region must embrace a gender-inclusive approach to achieve access and equity. The involvement of men challenges existing gender roles that relegate cervical cancer prevention to women and help reinforce current efforts to achieve cervical cancer elimination. It is important to emphasise that including men in cervical cancer prevention efforts does not entrench male dominance and diminish efforts to achieve gender equality but rather a fulfilment of the missing pillar in cervical cancer prevention in SSA. It is essential to conduct further research to understand men’s perspectives on their involvement in cervical cancer prevention. To achieve the elimination of cervical cancer in Sub-Saharan Africa, we must promote a comprehensive approach that includes men as vital stakeholders. This collaboration is key to ensuring access to healthcare and achieving health equity.

While fostering male engagement in prevention efforts is important, it is crucial to ensure a strong level of political will and commitment to funding the health system. Rwanda serves as an example of a country working toward the elimination of cervical cancer, as its increased health expenditures over the years have put it in a strong position to achieve universal HPV vaccination, making it the only African country to accomplish this (9). Additionally, local ownership of preventive health interventions significantly enhances the effectiveness of cervical cancer prevention efforts, particularly through collaborative initiatives involving women, community health workers, and local leadership (10).
